# Web based research in sexual medicine: a position statement of the European Society for Sexual Medicine

**DOI:** 10.1093/sexmed/qfad032

**Published:** 2023-07-05

**Authors:** Paraskevi-Sofia Kirana, Ahmet Gudeloglu, Andrea Sansone, Ioannis Sokolakis

**Affiliations:** Institute for the Study of Urological Diseases, Thessaloniki 54640, Greece; Department of Urology, Hacettepe University Hospital, Ankara, Turkey; Department of Experimental Medicine, Sapienza University of Rome, Rome, Italy; 2nd Department of Urology, Aristotle University of Thessaloniki, Thessaloniki 54640, Greece

**Keywords:** sexual medicine, web, surveys, research, statements

## Abstract

**Background:**

Although the use of the Web has brought major advances in every step of the research process, this also comes with several methodological challenges.

**Aim:**

The article presents the European Society for Sexual Medicine's position statements on key methodological concerns relative to Web-based research in sexual medicine.

**Methods:**

The authors conducted a systematic scoping review of articles using Web-based research methods in sexual medicine. For the creation of the statements, the authors processed the data from the methodology of the studies and formulated the final statements reaching 100% agreement in the group.

**Outcomes:**

European Society for Sexual Medicine statements were provided on the following domains: definition of the population of interest, selection of the population of interest, data collection quality, response rate, self-reported questionnaire, consent, and legal obligations.

**Results:**

Researchers should justify the relevance of the Internet population to the population of interest; should clearly describe how they identified study participants; should select and employ specific measures to avoid hoax; should explicitly describe the process of calculation of response and completion rates as well as the relative implications; should validate traditional sexual health questionnaires for online and, if possible, multilingual use; should not ignore consent in Web-based research; and need to be knowledgeable of the technical measures and legal obligations to protect anonymity.

**Implications:**

Researchers are advised to include trained computer scientists in their group, have a good understanding of their legal obligations as to collecting, storing and disseminating personal data, and design their studies by taking into account the challenges of Web-based research.

**Strengths and limitations:**

The heterogeneity of the included studies and methodological low quality of most of them was a limitation, which also shows the importance of this study and the need for guidelines regarding Web-based research.

**Conclusion:**

Large uncontrolled samples could be a threat to the quality of the studies and increase bias if researchers are not mindful of the methodological challenges they would need to account for.

## Introduction

Technological advancements have significantly changed the scientific research process, including collecting, storing, and analyzing data. For example, researchers can now easily access vast amounts of information from a variety of sources, including online databases, digital libraries, and social media platforms that are worldwide, rather than local. Large amounts of information, such as research data, hospital data, demographic data, etc., can be stored with cloud computing, thus switching from the limitations of paper and CD-ROMs to large storage spaces of low cost. Big data analytics permit the analysis of large datasets that would otherwise be impossible. In addition, collaborative platforms allow researchers based in different parts of the world to share data and findings, work together, and thus easily formulate research groups that are not limited geographically.

It is quite evident that digital technology is opening a window of great opportunity and potential for the development of sexual medicine research. For example, Web-based surveys can have the potential to reach large groups across the world saving both time and money while increasing more honest responding. Another example of great opportunity is Web-based qualitative research for hard-to-reach populations.^1^ It is now possible to conduct focus groups through video conversations with people from almost every part of the world sharing a common rare sexual behavior. Qualitative data can also be collected by observation of forums and cyber community chat rooms. Another example of the great opportunities that arise is the analysis of enormous amounts of data, such as Google searches, tracking cookies and Facebook users’ data. These are just a few examples that give us an idea of the opportunities of Web-based research.

At the same time, Web-based research goes with great challenges, as scientists may not be mindful of the relevant methodological disadvantages that influence the interpretation of the results.^2^ Key concerns that arise with Web-based research are Internet demography, self-selection, calculating response and completion rates, scale validity for online use, issues of informed consent, and anonymity.^2-4^ Suggestions to overcome the methodological challenges that are specific to Web-based surveys have been described in the literature, but researchers may not be familiar with these, in addition to the fact that some require specific information technology knowledge.

In a previous article, the European Society for Sexual Medicine (ESSM) provided a specific position statement in order to increase awareness on the needs and opportunities related to the use of digital technology in sexual medicine.^5^ Following the latter research line, ESSM developed this statement proposal, through a systematic scoping review of the literature, to raise awareness on key challenges of Web-based sexual medicine research and to encourage relevant scientific societies to respond to the emerging needs. Here, we present the ESSM’s position statements on key methodological concerns relative to Web-based research in sexual medicine.

## Method

### Systematic review of the literature

Using Cochrane’s methodological recommendations on systematic reviews, we conducted a systematic scoping review of the literature.^6^ Scoping studies comprise a further type of literature review that tends to address broader topics in which many different study designs might be applicable.^7^ Furthermore, scoping studies do not assess the quality of included studies, as they may have different designs.^8^ Until the end of 2021, we performed systematic research in the following databases: MEDLINE and the Cochrane Library. The keywords “online research” OR “online questionnaire” OR “search engine” OR “big data” OR “social media” OR “registry” OR “Web” OR “internet” were searched in combination with (AND) the terms “sexual” OR “erectile” in the title or abstract. Additionally, the reference lists were tracked backward for further relevant articles, which were not identified during the research. Furthermore, we reviewed articles that were suggested by the related citations in PubMed option for the most recent articles. Our research was restricted by including only articles in English language. There was no chronological restriction.

After screening the title and the abstract, all articles dealing with Web-based digital technology as research tools that were applied in sexual medicine research were included for full text reviewing. The screening of full articles was conducted by 4 reviewers (P.-S.K., I.S., A.S., and A.G.) independently with predefined exclusion criteria. Finally, any discrepancies were discussed between the reviewers, in order to reach a consensus. If a disagreement occurred, another author (A.B.) was designated in order to reach a consensus. We included original research clinical articles with primary intervention the use of any Web-based digital technology as research tool in sexual medicine.

The exclusion criteria were the following: articles with conditions or populations that cannot be included in sexual medicine; articles that did not provide sufficient information about the research methodology or the tool used in the research; articles not in English language; conference abstracts, case reports or small case series (≤10 patients), narrative reviews, letters to the editor, animal studies, or editorial comments; abstracts only (no full text available); and gray literature (eg, reports of device manufacturers) without publication in a scientific journal.

### Data extraction and statement development

Four authors (P.-S.K., I.S., A.S., and A.G.) independently performed a 3-step parallel screening of title, abstract, and full text of all identified records based on our predefined selection criteria. Data regarding study and patient characteristics, study design, and methodology were retrieved from all included studies and tabulated in a Microsoft Excel spreadsheet. Information was extracted and used to develop consensus statements. The quality of the included articles was not assessed, as the clinical results and outcomes of the included studies were not used for the development of the statements.

Τhe creation of the statements relied on (1) literature on methodological and ethical issues of Web based studies, (2) the results of the systematic review on the methodology of the Web-based sexual medicine studies and (3) group discussions. Members of the group reviewed the draft statements and subsequently met (online meeting) as a group to discuss discrepancies and finalize a draft consensus document. This allowed members of the group to provide further clarification on some matters and present arguments in order to justify their viewpoints. The final statements (Table 1) reached 100% agreement within the group (results of the systematic research can be seen on the Supplementary File 1).

**Table 1:** The ESSM statements on Web-based research in sexual medicine.

No.	**Statement**	Comment	Level of evidence^a^
**Definition of population of interest**			
1	**Researchers should justify the relevance of the internet population to the population of interest.**	Internet demography is not representative of the population, which can threaten the validity of the study particularly in sexual medicine where age is a relevant factor.	4
**Selection of population of interest**			
2	**Self-selection is a key challenge that may hinder the generalizability of Web studies, so researchers need to wisely select how they will identify study participants and the specific procedures need to be clearly described.**	Studies that recruit participants through open websites and with little control over who receives access to the survey may reach large numbers of population in limited time and cost, but results may be biased.	4
**Data collection quality**			
3	**Multiple submissions and hoax may threat the reliability of the results, so researchers are strongly advised to select and employ specific measures to avoid them.**	Researchers will need to be especially cautious when using incentives.	4
**Response rate**			
4	**Researchers need to explicitly describe the process of calculation of response and completion rates as well as the relative implications.**	It is very difficult to calculate the response rate in open surveys because the number of people that saw the questionnaire (which is different to those that visited the site) is difficult to control.	4
**Self-reported questionnaires**			
5	**Research to validate traditional sexual health questionnaires for online use should be encouraged as well as adapted for multilingual use.**	Global studies may reach populations that are not English native speakers, thus making multilingual adaptations necessary.	4
**Consent**			
6	**Asking for consent should not be ignored in Web-based research*.***	Informed consent has been an issue of debate when it comes to big data studies because such studies use data derived from many large databases that often are not developed for research.	4
**Legal obligations**			
7	**Researchers need to be knowledgeable of the technical measures and legal obligations to protect anonymity.**	Web researchers must take precautions to keep data protected against hacking, accidently leaking, or careless disclosure.	4
**Online randomized controlled studies**			
8	**Online randomized controlled studies are feasible when the interventions or the assessment of the outcomes do not require face-to-face interaction between the subjects and the investigators.**	It is imperative that more such studies are implemented to measure and improve the efficacy of online therapeutic interventions.	4

a According to the Centre for Evidence-Based Medicine (http://www.cebm.net).

Due to limited evidence and a lack of good study quality, no recommendations as per the Oxford 2011 Levels of Evidence criteria were possible (all Level of Evidence 4).^9^ However, considering the relevance of the topic, ESSM statements provide a summary of the Society’s position and suggestions for further research.

### Aspects to be considered to perform a Web-based survey

In order to increase the quality of the studies, their reproducibility and to reduce selection bias, several aspects need to be clearly explicated and clarified. In the following sections, specific suggestions and recommendations are provided.

### Definition of population of interest

#### Statement 1: researchers should justify the relevance of the Internet population to the population of interest

##### Evidence

Demography of the Internet population is not representative of the society population. Internet use is associated with younger age, male sex, and higher education levels, and there is variance in the type of Internet activity and the devices used by different subgroups.^10-12^ Whether the research is suitable for the Internet population is one of the first questions to be asked. Does the sample of interest use the Internet^2^? For example, one of the studies we reviewed collected Google trends data to analyze the web searches for sexual dysfunctions including Peyronie’s disease, erectile dysfunction, and premature ejaculation.^13^ However, the age of the Internet users was not reported by Google trends, and therefore there could be a significant bias in the type of information being sought, as older men may talk with their doctors instead of searching the Internet, whereas younger men are more likely to search the Internet.^13^

##### Remarks

The Internet demography is not representative of the population and may not be representative of the population of interest. This may threaten the generality of the results. In sexual medicine, with many conditions of interest being associated with age, the lower use of Internet by older populations needs to be taken into account when selecting the Web as a research tool.

### Selection of population of interest

#### Statement 2: self-selection is a key challenge that may hinder the generalizability of Web studies, so researchers need to wisely select how they will identify study participants and the specific procedures need to be clearly described

##### Evidence

Self-selection or the “volunteer effect” refers to the fact that people are more likely to respond to a questionnaire when they have special interest to the topic. For example, people responding to a questionnaire on sexual health may be more likely to respond if they are affected by a sexual problem or if the incentives are of special interest.^14^ As people who respond to the survey have different characteristics to those that do not respond, the results may be biased. For this reason, open surveys that recruit participants from websites or newsgroups provide more exploratory or preliminary data compared with those that recruit through email lists or through invitation only webpages. The latter have a more rigor and controlled population.^2^ On the other hand, open surveys may also generate interesting data (even if they are not necessarily generalizable), if qualitative analysis and/or hypothesis generation is the aim, or if the objective is to study trends over time.

Only 82 (63%) studies included in our review reported how the sample was recruited. Of those, 45 recruited the sample through direct invitation either using email or social media, while 47 studies recruited the sample through open websites. Only a few studies described or reported which websites referred participants to the study survey (see Supplementary File 1).

##### Remarks

Self-selection bias is a key challenge for Web-based studies. Researchers need to consider this when deciding how they will identify their participants. Studies that recruit participants through open websites and with little control over who receives access to the survey may reach large numbers of population in limited time and cost, but results may be biased. On the other hand, in studies that distribute their survey on a sample with known specific characteristics, the researchers have more control over their selection, and the results may have stronger external validity. The researchers need to pay attention to report on the procedure they used in order to identify participants.^15^

### Data collection quality

#### Statement 3: multiple submissions and hoax may threat the reliability of the results, so researchers are strongly advised to select and employ specific measures to avoid them

##### Evidence

Another problem that affects the reliability of the results is that a participant who is very eager to skew the results toward a preferred direction may answer a questionnaire multiple times. In the field of sexual health, rates of multiple submission vary from 8% to 33%.^16^ Approximately half of multiple submission were from subjects that participated 11 to 67 times.^16^ In a study conducted on men having sex with men, 4 categories of repeat responders were identified: infrequent (2-5 submissions), persistent (6-10 submissions), very persistent (11-30 submissions), and hackers (more than 30 submissions).^17^ One way that has been suggested to identify unique participants and to avoid multiple same participant entries is to use cookies.^2^ If cookies are used, it should be openly stated together with the fact that they are set to expire on the date the study will finish.^3^ Also, measuring response time has also been suggested as a way to identify hoax. For example, a very fast response time could be an indication of fraud, and these respondents could be excluded.^3^ IP addresses have been used to identify small variations to IP and infrequent responders. Several ways to avoid multiple completion and fraud have been described and analyzed, but it is beyond the scope of this article to describe them.^16^^,^^17^ In the studies that we reviewed, we found only 2 studies reporting using cookies or another method to reassure the uniqueness of each participant.^18^^,^^19^

##### Remarks

Data collected through the Internet is susceptible to multiple submissions and fraudsters, which may hack a study’s reliability. Researchers will need to employ measures to avoid this and be especially cautious when using incentives. The Web is evolving fast, and it will be necessary to have good programmers and staff who are knowledgeable of ways to avoid multiple responses and hackers.

### Response rate

#### Statement 4: researchers need to explicitly describe the process of calculation of response and completion rates as well as the relative implications

##### Evidence

The response rate is the number of people who answered the survey divided by the number of people in the eligible sample who saw the questionnaire (not just the website). The response rate can only be calculated with a defined sample group, such as a contact list or record of the number of people being approached to take the survey. For example, a survey that was administered via email would be able to calculate the response rate by knowing the number of people that opened their email and should not include the bounced emails. In open surveys, it is very difficult to control the number of people that saw the questionnaire. Unfortunately, methods like pop-ups and website embeds make it difficult to define the number of people who saw the survey and can therefore render any measurement of a response rate unreliable. Counting everyone who visited the webpage as invited to fill in the survey may lead to very low response rates. Other technical ways to identify these numbers, possibly with cookies or log file analysis, are required.^3^ The response rate in the surveys we reviewed ranged from 4% to 96% and was higher in surveys offering incentives or in special populations, although it was reported in <40% of the studies.

Different to the response rate is the completion rate, which refers to the number of surveys filled out and submitted divided by the number of surveys started by respondents. In other words, only the respondents who have actually entered the survey would be included in this statistic, and only those respondents who completed the full survey would increase the completion rate. Because it does not rely on the number of people contacted and is strictly based on people’s interaction with the survey, a completion rate can, and should, be measured on any survey, including email, intercept, pop-up, embedded, and hybrid surveys. A low completion rate is associated with longer surveys and more difficult questions.^20^ Unfortunately, in our review of surveys, completion rates were rarely reported. It could be that response rates and completion rates were in some cases used interchangeably, thus making it unclear what the rates refer to.

##### Remarks

Researchers are advised to report the response rate and whether it was sufficient to enable generalizing the results to the target population, how was it calculated, and what the potential nonresponse bias was.^15^ Relative implications for the interpretation of the results need to be described.

### Self-reported questionnaires

#### Statement 5: research to validate traditional sexual health questionnaires for online use should be encouraged as well as adapted for multilingual use

##### Evidence

An important concern is whether data captured in Web surveys are reliable and valid. Several studies are required to establish the validity of a scale. Simply translating the format from paper to the Web may lead to changes to what the questions and answers mean and therefore influence the validity of the survey.^21^ In our review, we found only 8 studies reporting the use of scales that were validated for online use.^7^^,^^22-28^ The International Index of Erectile Function online version was used in most cases. Another issue of concern is that global studies may reach populations that are not English native speakers. Therefore, questions that arise are whether the English versions are acceptable and whether multilingual online versions are available.

##### Remarks

Nowadays, the gold standard questionnaires in sexual medicine are the International Index of Erectile Function and the Female Sexual Function Index, and they have only recently been validated for online use.^29^^,^^30^ There is a need for (1) more studies to validate sexual health questionnaires for online use, (2) researchers to prefer using validated online versions, and (3) multilingual versions to be made available for large global studies.

### Consent

#### Statement 6: asking for consent should not be ignored in Web-based research

##### Evidence

Informed consent as a basic ethical tenet of scientific research on human populations should not be ignored in Web-based research. Informed consent is required when data are collected from research participants through any form of communication, interaction, or intervention or when behavior of research participants occurs in a private context in which an individual can reasonably expect that no observation or reporting is taking place. Informed consent is not required when researchers do research in public places or use publicly available information about individuals.^31-33^ While the participants of online questionnaire-based research have the choice whether to complete the survey, the observation of natural conversations in real-time chat rooms has serious ethical considerations associated regarding invasion of privacy. A question that arises is whether the Web space analyzed is a “public place” or a private space. For example, should observation studies on websites or narratives and/or interactions in newsgroups, mailing lists, chat rooms require participants to provide informed consent?^34^ Also, if the group moderator or administrator of the website provides informed consent, can it replace that of each individual participant? In addition, if the researchers post an announcement to a mailing list or newsgroup saying that it will be monitored and analyzed for the next few months, this may not only bias the results, but also damage the community by provoking many members to opt out. Another option is to retrospectively ask participants to provide consent for their data to be used. In other words, instead of asking for consent at the beginning of the study, asking for consent to disseminate the information. Technological advancements have significantly changed the scientific research process, including collecting, storing, and analyzing data. For example, researchers can now easily access vast amounts of information from a variety of sources, including online databases, digital libraries, and social media platforms that are worldwide, rather than local. Large amounts of information, such as research data, hospital data, demographic data, etc., can be stored with cloud computing, thus switching from the limitations of paper and CD-ROMs to large storage spaces of low cost. Big data analytics permit the analysis of large datasets which would otherwise be impossible. In addition, collaborative platforms allow researchers based in different parts of the world to share data and findings, work together, and thus easily formulate research groups that are not limited geographically.

Although this approach is time consuming, it is less intrusive.^35^ Informed consent has been an issue of debate when it comes to big data studies because such studies use data derived from many large databases that often are not developed for research. Questions as to whether informed consent stands as an obstacle to getting easy access to new knowledge or whether it is still required even if the means to get it needs improvement have attracted a lot of attention and have raised ethical concerns relative to big data health research.^36-38^

##### Remarks

We would like to emphasize that although getting informed consent is important, asking for consent is even more important. By asking for consent, the participant’s autonomy and dignity are being respected. This will have important long-term implications in the relationship formed between citizens and scientists. It is about nurturing a relationship of respect and trust.^39^ Even in cases were researchers have legal right to use one’s data, asking for consent indicates respect toward the subject’s autonomy and presumes an effort to commit to research from the subject as well. In addition, new ways to provide information about the study by using videos or attractive graphics have been suggested and could possibly make the informed consent a more attractive process.^40^

### Legal obligations

#### Statement 7: researchers need to be knowledgeable of the technical measures and legal obligations to protect anonymity

##### Evidence

Concerns about anonymity and security could decrease response rates. Participants may be reluctant to participate if anonymity is not clearly stated. This is especially important for sensitive items, such as sexual preferences and behaviors in which respondent anonymity may encourage participation and lower social desirability. When designing anonymous online research, the information collected must not identify and must not be used, either alone or with other information, to identify an individual. Examples of personal data according to the General Data Protection Regulation are a name and surname; an email address such as name.surname@company.com; an identification card number; location data (for example the location data function on a mobile phone); an Internet Protocol (IP) address; and a cookie ID.^41^ The Internet holds various pitfalls for researchers, who can easily and unintentionally violate the privacy of individuals.

- When conducting an anonymous survey online, the researcher must ensure that the hosting system does not link or associate with the survey data any information automatically passed to it from a survey participant’s device or computer that may be used to identify the individual.
- When an online survey provider is used to conduct survey research that involves personal information, it is the researcher’s responsibility to ensure that the provider’s terms of service agreement and privacy policy allow for the secure collection, retention, use, disclosure, security and disposal of personal information in accordance with the study policy.
- In qualitative studies, by quoting the exact words of a newsgroup participant, a researcher may breach the participant's confidentiality even if the researcher removes any personal information. This is because powerful search engines such as Google can index newsgroups (groups.google.com), so that the original message, including the email address of the sender, could be retrieved by anybody using the direct quote as a query.

While anonymous surveys may be ideal from a privacy perspective, they present several research challenges. One challenge is that, as there is no direct way of knowing who has responded to an anonymous survey, it will be difficult to follow up with those individuals who do not respond. A lack of follow-up could result in a poor response rate and, consequently, the validity of the results of the survey could be questioned. In addition, respondent anonymity and associated disinhibition may promote multiple submissions, especially when incentives are offered.^17^ “Fraudsters” can put a study’s generality and reliability in danger.^16^

##### Remarks

Web researchers must take precautions to keep data protected against hacking, accidently leaking, or careless disclosure.^42^ Researchers would need to be advised on the technical and also the legal requirements. For example, in Europe a General Data Protection Regulation expert would be required to ensure data protection.

### Online randomized controlled studies

#### Statement 8: online randomized controlled studies are feasible when the interventions or the assessment of the outcomes do not require face-to-face interaction between the subjects and the investigators

##### Evidence

In a wholly Internet-based randomized trial, the investigators never meet participants—neither the application of the intervention nor the assessment of the outcomes requires face-to-face interaction between the subjects and the investigators. A systematic review of randomized controlled trials (RCTs) of any health intervention conducted fully or primarily on the Internet was carried out and 23 fully and 27 primarily Internet-based RCTs were identified.^10^ In our review, we found 5 RCTs, 3 uncontrolled clinical trials, 1 case-control study, and 1 follow-up of an RCT.^43-52^ All studies assessed sex therapy interventions that were asynchronous, including mostly email communication with therapists and educational modules.

##### Remarks

Although not many studies were online RCTs, the implementation of such studies is feasible, and more such studies should be encouraged. Especially today, with social restrictions being experienced globally, it is imperative that more such studies are implemented to measure and improve the efficacy of online therapeutic interventions.

### Limitations and future perspectives

Our study and the provided statements have limitations that need to be addressed in future research. The major limitation is the heterogeneity of the included studies and low quality on most of them. This limitation also shows the importance of this study and the need for guidelines regarding Web-based research. Another limitation is that most of the studies were surveys and cross-sectional studies. Only very few studies were RCTs or interventional studies. More studies are needed to draw recommendations and provide guidelines for interventional studies considering the unique characteristics of sexual medicine. With increasing familiarization of the population with Web-based technologies, Web-based studies would increase in the future and the included population samples will be more representative.

## Conclusions

Although in sexual medicine Web-based research studies are frequent, several methodological aspects need attention. Suggestions for researchers conducting online studies that may be considered before the initiation of the studies and after completion can be seen in Table 2. Scientific societies in sexual medicine have a key role to play in the future of Web-based sexual medicine research. It is important that key measures are taken to support researchers employing Web-based techniques. Sexual Medicine Societies could work together to encourage online researchers. Table 3 summarizes several key tasks that could be accomplished through joint ventures.

**Table 2:** Suggestions for researchers conducting online studies in sexual medicine.

Advice for researchers designing Web-based studies	Features of the methodology to be described
Include trained computer scientists in their group Have a good understanding of their legal obligations as to collecting storing and disseminating personal data and Design their studies by taking into account the challenges of Web-based research	Why were the internet population chosen for the study? How were study participants identified and referred to the study? What measures were employed to avoid multiple submissions How were response rates calculated? How was consent to participate in the study obtained? What measures were taken for anonymity to be protected?

**Table 3:** Tasks for Sexual Medicine Societies to encourage online research.

Sexual medicine societies may work together to Develop their own survey provider platforms that comply with the necessary technical and legal requirements and make these available to researchers Develop standards of best research practice, procedures and checklists that online researchers would need to follow when designing and reporting their Web study Publish guidelines to ensure ethical research practice Support researchers with the study design and sampling method Encourage validation of instruments for online use

Technological advances are evolving very fast and soon will bring us into an era of virtual reality, machine learning, and blending of offline and online worlds. We need to be ready to design studies that are prepared to accommodate new technologies.

## Supplementary Material

SupplementEK_qfad032
