# Supplementary material for: Web based research in sexual medicine: a position statement of the European Society for Sexual Medicine
Source: Sex Med. 2023 Jul 5;11(3):qfad032. doi: 10.1093/sexmed/qfad032 (PMC10324026; doi:10.1093/sexmed/qfad032)
Supplement: SupplementEK_qfad032 [file supplementek_qfad032.docx]

**Supplement 1**

**Results of the Systematic Research**

From the initial 2320 articles, after excluding the duplicates, non-English literature and abstracts only 1109 articles remained. After title and abstract screening 890 articles were excluded and 219 remained. After full-text screening 75 additional papers were excluded remaining 144 articles, which met our inclusion criteria (Figure 1). Most of the included studies (n=120) were web-based surveys or cross-sectional studies. On the other hand, five (5) studies were randomised controlled trials, two (2) uncontrolled clinical trials, two (2) used the social media as an intervention channel to spread sexual health messages, one (1) case-control study and one (1) follow-up of a RCT. Furthermore two (2) studies validated online questionnaires, three (3) were qualitative studies, five (5) studies analysed big-data and three (3) retrospective studies of online records.

As shown in the screening flow-chart we included 120 surveys and cross-sectional studies for the final analysis (**Figure 1**). The older paper was published in 2004 and was conducted in the United Kingdom (UK) [1]. The time period of the conducted studies ranged from 1 week to 15 months. The sample size ranged from 33 to 92,620 patients, with a median sample size of 650 patients. Only 2 studies reported using cookies or other methods to reassure the uniqueness of each participant [2,3]. Most of the studies (n=54) were conducted in the field of male sexual dysfunctions (including erectile dysfunction, premature ejaculation and Peyronie’s disease), followed by studies regarding sexuality and sexual function of both sexes (n=24), studies in sexual behaviour (n=24), sexual awareness (n=17) and one study in sexuality of the elderly. Many of studies were conducted in a special population like men having sex with men (MSM) (n=12), patients with prostate cancer (n=3), patients after pelvic fracture (n=1), and patients with bladder exstrophy syndrome (n=1). The majority of the studies used validated questionnaires (n=56), although only 8 studies reported or used questionnaires that were validated for web-based research (mostly IIEF; International Index of Erectile Function). The response rate in the surveys ranged from 4% to 96% and was higher in surveys offering incentives or in special populations, although it was reported in less than 40% of the studies. About half of the studies (n=45) recruited the sample size through direct invitation either using email or social media, while 47 studies recruited the sample size through open web sites. The dropout rates were rarely reported in the studies. The included RCTs and interventional studies used as intervention the online sexual therapy or online consultation [4–8]. Although with methodological weaknesses, these studies showed the feasibility of online psychosexual treatment in sexual medicine (mostly psychogenic ED). Two recent studies used Big Data (Google trends and Twitters tweets respectively) to investigate male sexual dysfunction [9,10].

**Figure 1: Flow chart of the screening process and the inclusion of the articles**


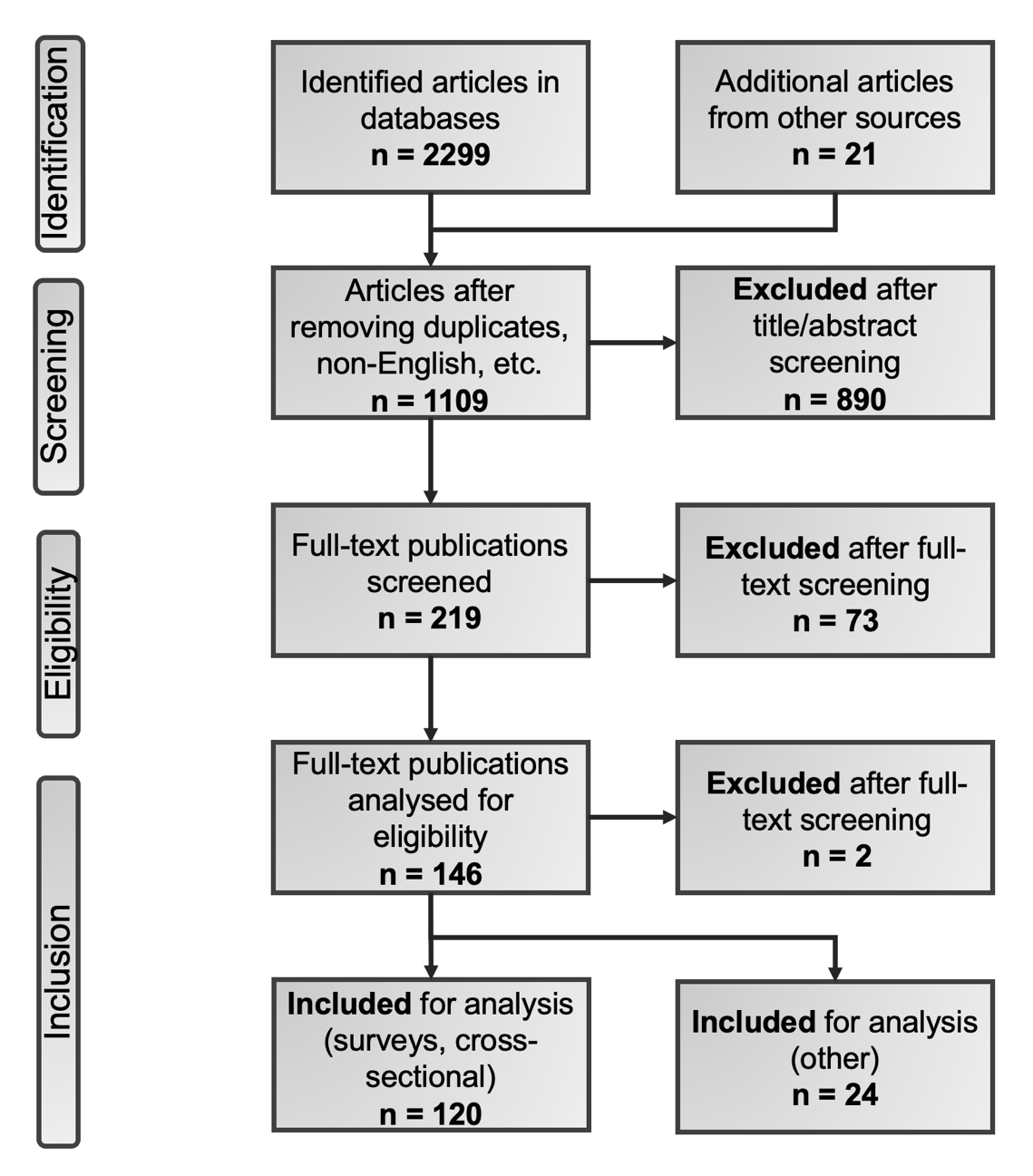


**REFERENCES**

[1] Eardley I, Dean J, Barnes T, Kirby M, Glasser D, Solanki J. The sexual habits of British men and women over 40 years old. BJU Int März 2004;93:563–7.

[2] Hirshfield S, Chiasson MA, Wagmiller RL, Remien RH, Humberstone M, Scheinmann R. Sexual dysfunction in an Internet sample of U.S. men who have sex with men. J Sex Med Sept 2010;7:3104–14.

[3] Grabski B, Kasparek K, Muldner-Nieckowski L, Iniewicz G. Sexual Quality of Life in Homosexual and Bisexual Men: The Relative Role of Minority Stress. J Sex Med Juni 2019;16:860–71.

[4] Andersson E, Walén C, Hallberg J, Paxling B, Dahlin M, Almlöv J. A Randomized Controlled Trial of Guided Internet‐delivered Cognitive Behavioral Therapy for Erectile Dysfunction. J Sex Med 2011;Oct;8(10):2800–9.

[5] Blanken I, Leusink P, van Diest S, Gijs L, van Lankveld JJ. Outcome predictors of Internet-based brief sex therapy for sexual dysfunctions in heterosexual men. J Sex Marital Ther 2015;41:531–43.

[6] van Lankveld J, Leusnik P, van Diest S, Gijs L, Slob A. Internet-based brief sex therapy for heterosexual men with sexual dysfunctions: a randomized controlled pilot trial. J Sex Med 2009;Aug;6(8):2224–36.

[7] Schover LR, Canada AL, Yuan Y, Sui D, Neese L, Jenkins R. A randomized trial of internet-based versus traditional sexual counseling for couples after localized prostate cancer treatment. Cancer 2012;15;118(2):500–9.

[8] Van Diest SL, Van Lankveld JJ, Leusink PM, Slob AK, Gijs L. Sex therapy through the internet for men with sexual dysfunctions: a pilot study. J Sex Marital Ther 2007;Apr;33(2):115–33.

[9] Russo GI, di Mauro M, Cocci A, Cacciamani G, Cimino S, Serefoglu EC, et al. Consulting “Dr Google” for sexual dysfunction: a contemporary worldwide trend analysis. Int J Impot Res 2020;32:455–61. https://doi.org/10.1038/s41443-019-0203-2.

[10] Sansone A, Cignarelli A, Ciocca G, Pozza C, Giorgino F, Romanelli F. The Sentiment Analysis of Tweets as a New Tool to Measure Public Perception of Male Erectile and Ejaculatory Dysfunctions. Sex Med 2019;Dec;7(4):464–71.
